# Insulin, Glucose and Lipid Biomarkers as Possible Risk Factors for Functional Somatic Disorder: A Five-Year Follow-Up of the DanFunD Cohort

**DOI:** 10.3390/jcm15155793

**Published:** 2026-07-24

**Authors:** Torben Jørgensen, Rikke Kart Jacobsen, Sine Wanda Jørgensen, Marie Weinreich Petersen, Anne Ahrendt Bjerregaard, Lise Kirstine Gormsen, Tina Birgitte Wisbech Carstensen, Signe Ulfbeck Schovsbo, Line Lund Kårhus, Niklas Rye Jørgensen, Allan Linneberg, Thomas Meinertz Dantoft

**Affiliations:** 1 Center for Clinical Research and Prevention, Copenhagen University Hospital–Bispebjerg and Frederiksberg, 2000 Copenhagen, Denmark; rikke.kart.jacobsen@regionh.dk (R.K.J.); anne.ahrendt.bjerregaard@regionh.dk (A.A.B.); signe.ulfbeck.schovsbo@regionh.dk (S.U.S.); line.lund.kaarhus@regionh.dk (L.L.K.); allan.linneberg@regionh.dk (A.L.); thomas.meinertz.dantoft@regionh.dk (T.M.D.); 2 Department of Public Health, Faculty of Health and Medical Science, University of Copenhagen, 1353 Copenhagen, Denmark; 3 Department of Cardiology and Endocrinology, Holbæk Hospital, 4300 Holbæk, Denmark; siwj@regionsjaelland.dk; 4 Department of Functional Disorders, Aarhus University Hospital, 8200 Aarhus, Denmark; mawept@rm.dk (M.W.P.); lisegorm@rm.dk (L.K.G.); tinacars@rm.dk (T.B.W.C.); 5 Department of Clinical Medicine, Aarhus University, 8200 Aarhus, Denmark; 6 Department of Clinical Medicine, Faculty of Health and Medical Science, University of Copenhagen, 2200 Copenhagen, Denmark; niklas.rye.joergensen@regionh.dk; 7 Department of Clinical Biochemistry, Rigshospitalet, 2100 Copenhagen, Denmark; 8 Translational Research Centre, Rigshospitalet, 2100 Copenhagen, Denmark

**Keywords:** functional somatic disorder, longitudinal study, epidemiology, incidence, lipids, glucose, insulin

## Abstract

**Background**: Functional somatic disorder (FSD) is prevalent, but pathological pathways have not yet been identified. Cross-sectional studies suggest an association between FSD and lipid and glucose metabolism. The aim of the present prospective study was to assess whether metabolic factors represent possible risk factors for development of FSD. **Methods**: A population-based cohort of 9656 men and women aged 18–76 years was established in 2011–2015, and 5738 participated in a re-examination after a median of 65 months. At both baseline and follow-up, participants answered questionnaires on lifestyle and various delimitations of FSD including bodily distress syndrome (BDS), chronic fatigue (CF), chronic widespread pain (CWP), irritable bowel (IB), and multiple chemical sensitivity (MCS). Fasting values of cholesterol, triglycerides, glucose, HbA1c, insulin, and insulin resistance were assessed at baseline and analyzed in relation to incidence of FSD using logistic regression models, adjusted for age, sex, lipid- and glucose-lowering medications, obesity, social status, lifestyle, and sleep quality. **Results**: Several small, although significant, effects were observed. Lower levels of cholesterol were associated with development of BDS (OR: 0.982 (95% CI: 0.971–0.993)), CF (OR: 0.980 (95% CI: 0.964–0.996)), and CWP without co-morbid FSS among men. Higher levels of triglycerides were associated with development of CF (OR: 1.022 (95% CI: 1.003–1.041)). Glucose and HbA1c levels were not associated with development of FSD. Insulin and HOMA-IR levels were associated with development of CF in a non-linear relation, being negative at lower levels and changing to a positive relation at higher levels. **Conclusions**: More large-scale cohort studies are necessary to explore whether metabolism could play a role in the pathological pathway of FSD as indicated in the present study.

## 1. Background

Functional somatic disorder (FSD) is a diagnosis encompassing overlapping disorders, including the diagnostic construct bodily distress syndrome (BDS) and various functional somatic syndromes (FSSs) such as irritable bowel syndrome (IBS), fibromyalgia or chronic widespread pain (FM/CWP), chronic fatigue syndrome (CFS), and multiple chemical sensitivity (MCS). Common to these conditions are patterns of persistent and troublesome physical symptoms accompanied by impairment that cannot be better explained by other physical or mental disorders [1]. As no specific biological or physiological tests have yet been identified to establish a diagnosis, delimitations depend on the presentation of characteristic symptom patterns. In research many of these approaches lack standardization, which potentially gives rise to inconsistent results in the literature. Furthermore, a considerable overlap is seen between the various FSDs with many persons meeting the criteria for multiple delimitations simultaneously [2].

The literature points at chronic stress as a predictor for development of FSD [3,4]. Chronic stress (mental, social, or physical) can lead to alteration in the hypothalamic–pituitary–adrenal (HPA) axis, which is central to the construct of allostatic load, a physiological measure of the cumulative wear and tear of the body due to repeated cycles of attempts to adapt to changes. Several effects of an allostatic load have been described, one of them being impaired metabolic changes [5]. In the Danish Study of Functional Disorders (DanFunD) [6], we showed an association between FSD and lipid and glucose metabolism [7,8,9]. As cross-sectional studies can rarely distinguish causes from consequences, prospective studies are needed. A systematic review identified few cohort studies examining plasma lipids and the development of FSD with divergent results [10]. Another cohort study [11] showed that a lifetime diagnosis of high cholesterol was associated with development of self-reported CFS. These studies did not focus on lipid metabolism and FSD but mostly dealt with data from national registries. To the best of our knowledge, no longitudinal studies have addressed whether plasma glucose or insulin levels are associated with development of FSD. A small case–control study hypothesized that insulin resistance may underlie pathological mechanisms leading to central pain [12]. This hypothesis was supported by a large observational study showing less chronic pain among metformin users [13] and by clinical and experimental studies demonstrating effects of metformin on both chronic pain and fatigue [14,15].

This calls for a closer assessment of the role of lipid and glucose metabolism in the development of FSD. The aim of the present study was to investigate the role of plasma cholesterol, triglycerides, glucose, glycated hemoglobin (HbA1c), insulin, and insulin resistance in the development of FSD using the five-year follow-up investigation of the DanFunD study population.

## 2. Methods

### 2.1. Study Population

The DanFunD baseline cohort was established in 2011–2015 and consists of a part 1 (n = 2163) and a part 2 (n = 7493) [6]. DanFunD part 1 was conducted as a 5-year follow-up investigation of the Health2006 cohort carried out in 2011–2012. DanFunD part 2 was a new random sample selected through the National Civil Registration System and carried out in 2012–2015 among people living in the same 10 municipalities in the western part of greater Copenhagen, Denmark, as persons recruited to Health2006. Thus, a total of 9656 participants (33.7% of the invited) constituted the DanFunD baseline cohort. The participants were all born in Denmark and were between 18 and 76 years of age. All participants completed a self-administered paper-based questionnaire and participated in a general health examination conducted by trained nurses at the Research Centre for Prevention and Health (now: Centre for Clinical Research and Prevention).

In 2017–2020, all eligible participants from baseline (n = 9323) were invited for a follow-up study, and a total of 5738 (61.5%) participated. The self-administered questionnaires at follow-up were filled electronically, but only participants of DanFunD part 2 were invited for a general health examination conducted by trained nurses. The median follow-up period was 65 months (range 47–108 months). At both health examinations, participants were instructed to fast for at least six hours and avoid smoking for at least one hour beforehand.

FSD was assessed at baseline and follow-up. Using validated questionnaires [16], delimitation of FSD was operationalized using two different but overlapping approaches, i.e., the unifying diagnostic construct of BDS and FSS based on bothersome symptoms within the last 12 months. BDS was measured using the BDS checklist [17], comprising 25 symptoms across four symptom clusters: cardiopulmonary, gastrointestinal, musculoskeletal, and general symptoms. Based on the number of involved symptom clusters, BDS was classified as single-organ BDS (symptoms within one or two of the symptom clusters), multi-organ BDS (symptoms from at least three of the symptom clusters), or total BDS (either single-organ or multi-organ). In the FSS approach, four delimitations were used: irritable bowel (IB) [18]; chronic fatigue (CF) [19]; chronic widespread pain (CWP) [20,21]; and multiple chemical sensitivity (MCS) [22,23,24]. In these delimitations the word syndrome is avoided due to current disagreements on terminology and criteria. Persons with IB, CF, CWP, or MCS without any of the three other delimitations (i.e., without co-morbid FSS) were assessed in separate analyses [2]. Algorithms for delimitation of the various FSDs have been previously published [16].

Incident cases were defined as participants who did not fulfil the criteria for a given delimitation at baseline, but did fulfil them at follow-up. Incidence was assessed separately for BDS and for each of the four FSSs.

### 2.2. Assessment of Biomarkers

Fasting blood samples were drawn during the health examination and total cholesterol, HDL cholesterol, and triglycerides were measured from freshly collected fasting plasma samples using colorimetric slide methods with Vitros 4600/5600 Ortho Clinical Diagnostics (OCD), Rariton, NJ, USA. Non-HDL cholesterol was calculated by subtracting HDL cholesterol from total cholesterol. Fasting plasma samples were analyzed for glucose and insulin and whole blood was analyzed for glycated hemoglobin A1c (HbA1c). Glucose was assessed using hexokinase/glucose-6-phosphate dehydrogenase assay (Roche Diagnostics, formerly Boehringer Mannheim, Mannheim, Germany); insulin was assessed using the fluoroimmunoassay technique, AutoDelfia (Perkin Elmer-Wallac, Turku, Finland); and HbA1c was assessed using the HPLC method (TOSOH, Minato, Japan). The homeostasis model of insulin resistance, HOMA-IR, was calculated using the following formula: ([fasting plasma insulin (pmol/L)] × [fasting plasma glucose (mmol/L)]/22.5) × 0.144. This estimate can be used as a surrogate quantification of insulin sensitivity based on the concept that blood glucose and insulin concentrations are related by the feedback of glucose on beta cells to increase insulin secretion [25].

### 2.3. Covariate Assessment

Information on lifestyle factors was collected using validated questionnaires at baseline. Smoking status was categorized into “smoker”, “former smoker”, or “never smoker”. Alcohol consumption was measured as number of drinks per week (categorized into teetotallers (0); low to moderate intake (up to 14/21 units/week for women and men, respectively); and high (above 14/21 units/week). Dietary intake was estimated using a self-administered 26-item food frequency questionnaire resulting in a Dietary Quality Score classifying dietary quality into three categories (healthy, average, and unhealthy) [26]. Physical activity was assessed using the physical activity scale questionnaire adding the number of metabolic equivalents achieved during a common day [27]. Self-perceived social position was measured using a social 10-step staircase [28]. Sleep quality was assessed by two questions—“How often do you have difficulty falling asleep?” and “How often do you wake up too early?”—both with four response categories (once a month or more seldom; two to four times a month; one or more times a week; daily). Sleep quality was categorized as low (one or more times a week or daily for both questions), average (one or more times a week or daily for one of the questions) and high (once a month or more seldom or two to four times per month for both questions). Height and weight were measured once with the participant in light clothes without shoes and BMI (kg/m^2^) was calculated.

### 2.4. Statistics

Descriptive statistics (median and interquartile range) were used to characterize distributions of biomarkers among participants with and without FSD. Differences in the proportion of missing information in the metabolic measures between FSD and non-FSD were tested using chi square or Fisher’s exact test if the expected numbers were small. Associations between metabolic biomarkers and incident FSD were investigated using logistic regression models. Five FSDs served as dependent variables: BDS, CWP, CF, IB, and MCS. Eight primary exposures were examined: total cholesterol, non-HDL cholesterol, HDL cholesterol, triglycerides, glucose, HbA1c, insulin, and HOMA-IR. For each primary exposure, three hierarchically adjusted models were built. Model 1 included the primary exposure, sex, and age. For lipid analyses, use of cholesterol-lowering medication was additionally included as was use of glucose-lowering medications in the glucose and insulin analyses. Model 2 extended Model 1 by adding BMI and subjective social status. Model 3 further adjusted for lifestyle factors (smoking, alcohol intake, diet, and physical activity) and sleep quality. When sample size permitted, results from Models 1 and 3 were reported. Due to limited numbers of participants with incident multi-organ BDS and MCS, only Model 1 was used for these outcomes. For IB and CWP without co-morbid FSS, a modified version of Model 2 including sleep quality was applied in place of Model 3. Association between continuous variables and each FSD outcome was first modelled with restricted cubic splines (RCSs) with five knots at percentiles 5, 27.5, 50, 72.5, and 90 according to Harrell [29]. Stepwise model reduction (i.e., four knots at percentiles 5, 35, 65, and 95; three knots at percentiles 10, 50, and 90; linearity) was evaluated with the likelihood ratio test using a 5% significance level. For primary exposures, results regarding non-linear associations were presented modified in both tables and in figures. The modification was chosen to both reflect the optimal model and simplify interpretation of results by refitting the model assuming piecewise linearity with knots appropriately selected at either the median or the 10th and 90th percentile. Non-linear terms for secondary covariates were modelled using RCSs where relevant. Results are presented as ORs with 95% confidence intervals and interpreted as the impact of a one-unit increase in the primary exposure. Sex differences were examined in Model 1 by testing the interaction between each primary exposure and sex. When a significant interaction was found, the results were presented for each sex. All results were interpreted at a 5% significance level. Statistical analyses were performed using the logistic procedure in SAS Enterprise Guide version 8.4.2.22 (SAS Institute Inc., Cary, NC, USA).

## 3. Results

Among the participants at follow-up, 4881 and 4831, respectively, did not have BDS or any of the FSSs at baseline. After exclusion of participants who had not adhered to the fasting requirement, 4611 (52.2% women) and 4557 (51.8% women), respectively, were included in the analyses. At follow-up, 526 participants developed BDS (498 with single-organ BDS and 28 with multi-organ BDS), 240 developed CF, 172 CWP, 97 IB, and 26 MCS. The distribution of the metabolic parameters at baseline in the various FSD groups is shown in Table 1 and Table 2. Missing information on the primary exposures did not differ significantly between those with and without FSD. N-values in Table 1 and Table 2 differ a little due to missing data on lipid-lowering medications and glucose-lowering medications.

### 3.1. Cholesterol

Lower levels of total, non-HDL and HDL cholesterol were significantly associated with development of BDS (except multi-organ BDS) in the fully adjusted models (Table 3). The same trend was observed in development of CF (Table 4), being significant for total cholesterol and for HDL cholesterol in men. A similar trend was observed for CWP (Table 5), but not significant. There was a significant positive association between non-HDL cholesterol and development of IB (Table 6). Low HDL cholesterol was significantly associated with development of MCS (Table 7). In the analyses of the various FSSs without co-morbid FSS, non-HDL cholesterol became significantly negatively associated with development of CF (Table S2). In men, a significant negative association with development of CWP (Table S3) was observed, and the association between non-HDL cholesterol and IB disappeared (Table S4).

### 3.2. Triglycerides

Baseline triglyceride levels were not associated with development of BDS in the fully adjusted models (Table 3). As the association between baseline triglycerides and development of CF was not constant at all levels of triglycerides, associations are presented below and above the median value 1.0 mmol/L (Table 4). Among participants with triglycerides above 1.0 mmol/L, higher levels of triglycerides were associated with higher odds of CF. There was no association between triglycerides and development of CWP (Table 5) or IB (Table 6). Participants who developed MCS showed a trend towards higher baseline triglycerides, being significant for those with triglycerides below 1.0 mmol/L (Table 7), but the relation disappeared when participants with co-morbid FSS were removed (Table S5).

### 3.3. Glucose and HbA1c

Plasma glucose and HbA1c at baseline were not associated with development of BDS (Table 3). As the association between baseline glucose and development of CF was not constant at all levels of glucose, associations are presented below and above the median value of 5.4 mmol/L (Table 4). Low levels of glucose below 5.4 mmol/L seemed to be associated with development of CF in the fully adjusted model, but not for glucose values above 5.4 mmol/L. HbA1c was not associated with development of CF. Glucose and HbA1c were not associated with development of CWP or IB (Table 5 and Table 6). As regards development of MCS, the significant positive trend towards higher levels of glucose (Table 7) disappeared when participants with co-morbid FSS were removed (Table S5). HbA1c was not associated with development of MCS (Table 7).

### 3.4. Insulin and Insulin Resistance

**Figure 1:**
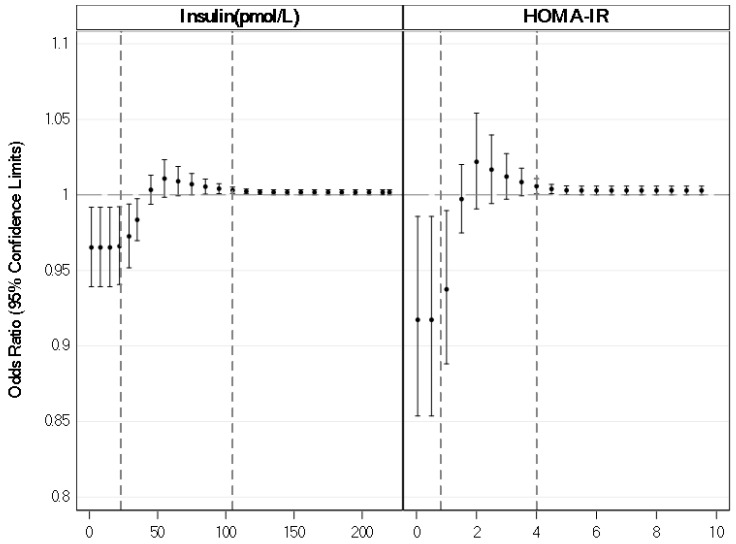
Odds ratios with 95% confidence interval of incident CF per 1-unit increase in levels of insulin and 0.1-unit increase in HOMA-IR fitted by logistic regression with restricted cubic splines with knots in percentiles 5, 35, 65, and 95. Vertical dashed lines indicate the 10th and 90th percentiles, which were used as the a priori breakpoints in the corresponding piecewise linear models reported in Table 4. The DanFunD study.

Insulin and HOMA-IR were not associated with the development of BDS in the fully adjusted model (Table 3). As the association between baseline insulin and development of CF was not constant at all levels of insulin, associations are presented below 23 pmol/L (10th percentile), between 23 and 104 pmol/L, and above 104 pmol/L (90th percentile). For insulin below 23 pmol/L, low insulin seemed to be associated with development of CF, whereas for insulin above 104 pmol/L, high insulin was associated with development of CF in the fully adjusted models. Likewise, the association between baseline HOMA-IR and CF was not constant at all levels of HOMA-IR. Lower levels of HOMA-IR, when below 0.8 units, were associated with development of CF in the fully adjusted model (Table 4), but the trend towards HOMA-IR levels above 4.0 being positively associated with development of CF was not significant. The complex relation between both insulin and HOMA-IR is also illustrated in Figure 1. Neither insulin nor HOMA-IR was associated with development of CWP, IB, and MCS in the fully adjusted models (Table 5, Table 6 and Table 7). Analyses of the various FSSs without co-morbid FSS (Tables S2–S5) did not change the results.

## 4. Discussion

This study represents the first large prospective epidemiological study analyzing insulin, glucose and lipid biomarkers in relation to developments of various delimitations of FSDs in a five-year follow-up period. Low cholesterol was associated with development of BDS, CF, and CWP (in men). Persons who developed CF showed association with high triglycerides and low glucose. CF was further associated with insulin and insulin resistance in a complex manner, where lower levels of these biomarkers were negatively associated, whereas higher levels were positively associated with development of CF. IB and MCS showed no association with either of the biomarkers when participants with co-morbid FFS were removed.

Our findings regarding lipid metabolism and development of FSD are not in accordance with the literature on the subject. However, the available sparse literature did not include direct measures of lipid metabolism, but merely represented more global measures of possible determinants of FSD [10,11]. To our knowledge, no other studies have analyzed the effect of glucose and insulin biomarkers on development of FSD in a prospective setting. Most of the literature on both lipid and glucose metabolism in relation to FSD comprises cross-sectional and case–control studies mostly conducted in clinical settings [12,30,31,32,33] and should be interpreted with care, as having FSD could lead to changes in lifestyle and thereby influence lipid and glucose levels. The present study confirms the findings from cross-sectional studies showing an association between insulin and insulin resistance and development of CF [9,30], but not with the studies showing an association between glucose metabolism and FM, CWP, and IBS [12,31,33]. Although our findings do not support the hypothesis that impaired glucose metabolism is a major risk factor for FM/CWP at the population level, they do not exclude a therapeutic role for metformin in established FM [13,15]; our findings support a therapeutic effect of metformin on fatigue in FM patients [14]. Metformin is used to improve glycemic control in diabetes by enhancement of insulin sensitivity in skeletal muscles. The discrepancies between the present study, cross-sectional studies and interventional studies could be due to the fluctuating status of FSD [34] meaning that a five-year period is too long for assessing an effect of insulin and insulin resistance on development of FSD. Further analyses using a shorter time period between repeated measurement of glucose metabolism and development of FSD are needed.

The present findings of low cholesterol levels being associated with the development of BDS, CF, and CWP (in men) after adjustment for BMI, social factors, lifestyle, and sleep quality are new and could point to a possible effect of cholesterol on development of FSD. It is well-known that high cholesterol is a risk factor for development of cardiovascular diseases and beneficial results from lowering cholesterol with statins are well established [35]. However, cholesterol has various beneficial effects in the body. Cholesterol is important for the structure of the cell membrane [36,37], and is a precursor for development of vitamin D, estrogen, testosterone, and adrenal hormones and thus has multiple functions in the human body. Our findings are in accordance with the well-known side effect of treatment with statins which can cause muscular pain [38].

### Strengths and Limitations

The current study has several strengths: First, we included a large (N = 5738) unselected sample from the general adult population with almost equal distribution of the two sexes. The population-based prospective study design reduced the risk of selection bias and allowed the results to be generalized to other adult populations. The prospective design also reduces the problem of whether we are dealing with predictors or consequences of these conditions. Second, the primary focus of the DanFunD study was to unravel the epidemiology of FSD, which is seldom the case for other studies in this field of research. Third, as many different criteria and delimitations for identifying FSD have been proposed, we included two approaches for defining FSD in our study. Hence, we attempted to capture the diverse nature of these conditions. In addition, by using various delimitations of FSSs, we succeeded in analyzing the effect of possible risk factors for cases without co-morbid FSS.

However, our study also has some limitations. First, the response rate of 33.7% at the baseline examination may be considered low, and even though the risk of selection bias is markedly reduced compared with clinical studies, we cannot completely rule it out. However, a former DanFunD study did not find a major problem with selection bias in relation to response rate [39]. Second, as the case definitions are based on questionnaires, we cannot rule out the possibility that some of the cases were misclassified [40]; hence, some of the obtained associations may have been caused by participants having diseases other than FSD. Third, as FSDs are fluctuating conditions [34], some incidence cases may represent recurrence rather than true new onset, potentially leading to misclassification of incidence. Forth, the many tests performed could lead to false positive findings due to multiple testing, but being an explorative study in a new field, we found it important to avoid type 2 errors.

## 5. Conclusions

In this large incidence study on the role of lipid and glucose metabolism in relation to development of FSD, we have shown for the first time that low cholesterol is associated with development of BDS, CF and CWP (men). Furthermore, insulin and insulin resistance may be involved in development of CF. More large-scale cohort studies are necessary to explore whether metabolism could play a role in the pathological pathway of FSD.

## Figures and Tables

**Table 1:** Distribution of total cholesterol, non-HDL cholesterol, HDL cholesterol, triglycerides, glucose, HbA1c, insulin, and HOMA-IR at baseline in persons who adhered to fasting and who developed bodily distress syndrome (BDS) (total, single-organ, and multi-organ) and did not (no BDS) at follow-up. Only persons without BDS at baseline were included (N = 4611). Thus, the numbers represent the cumulative incidence of BDS. The DanFunD study.

Lipid Metabolism	BDS			No BDS N = 4067 ^d^
	Total N = 524 ^d^	Single-Organ N = 496 ^d^	Multi-Organ N = 28 ^d^	
Total cholesterol ^a^ (N_missing_) Median (IQR)	(2) 5.20 (4.60–5.90)	(2) 5.20 (4.50–5.90)	(0) 5.35 (4.85–5.85)	(12) 5.30 (4.70–6.00)
Non-HDL cholesterol ^a^ (N_missing_) Median (IQR)	(2) 3.74 (3.07–4.47)	(2) 3.72 (3.04–4.47)	(0) 3.86 (3.54–4.43)	(12) 3.81 (3.16–4.55)
HDL cholesterol ^a^ (N_missing_) Median (IQR)	(2) 1.41 (1.18–1.65)	(2) 1.41 (1.18–1.65)	(0) 1.31 (1.14–1.61)	(12) 1.43 (1.18–1.74)
Triglycerides ^a^ (N_missing_) Median (IQR)	(2) 0.97 (0.73–1.48)	(2) 0.96 (0.72–1.48)	(0) 1.10 (0.88–1.49)	(12) 0.99 (0.74–1.41)
Glucose metabolism	Total N = 522 ^e^	Single organ N = 494 ^e^	Multi-organ N = 28 ^e^	N = 4068 ^e^
Glucose ^a^ (N_missing_) Median (IQR)	(5) 5.40 (5.10–5.80)	(5) 5.40 (5.00–5.80)	(0) 5.35 (5.20–5.75)	(26) 5.40 (5.10–5.80)
HbA1c ^b^ (N_missing_) Median (IQR)	(2) 5.40 (5.20–5.60)	(2) 5.40 (5.20–5.60)	(0) 5.40 (5.30–5.65)	(20) 5.40 (5.20–5.60)
Insulin ^c^ (N_missing_) Median (IQR)	(28) 50.8 (34.6–72.4)	(25) 51.0 (34.6–72.4)	(3) 45.0 (35.6–67.5)	(191) 46.3 (31.5–68.4)
HOMA-IR (N_missing_) Median (IQR)	(29) 1.74 (1.15–2.71)	(26) 1.76 (1.14–2.71)	(3) 1.54 (1.26–2.55)	(199) 1.61 (1.06–2.48)

HDL: high-density lipoprotein. HbA1c: % glycated hemoglobin; HOMA-IR: homeostatic model assessment for insulin resistance ((plasma insulin × plasma glucose/22.5) × 0.144). ^a^: mmol/L; ^b^: % glycated hemoglobin; ^c^: pmol/L; ^d^: only participants with information on lipid-lowering medications; ^e^: only participants with information on glucose-lowering medications.

**Table 2:** Distribution of total cholesterol, non-HDL cholesterol, HDL cholesterol, triglycerides, glucose, HbA1c, insulin, and HOMA-IR at baseline in persons who adhered to fasting and who developed functional somatic syndromes (FSSs), such as chronic fatigue (CF), chronic widespread pain (CWP), irritable bowel (IB), or multiple chemical sensitivity (MCS), and did not (no FSS) at follow-up. Only persons without FSS at baseline were included (N = 4557). Thus, the numbers represent the cumulative incidence of various FSSs. The DanFunD study.

Lipids	CF N = 239 ^e^	CWP N = 171 ^e^	IB N = 97 ^e^	MCS N = 26 ^e^	No FSS ^d^ N = 4.018 ^e^
Cholesterol ^a^ (N_missing_) Median (IQR)	(2) 5.00 (4.50–5.70)	(0) 5.10 (4.60–5.80)	(0) 5.10 (4.60–5.80)	(1) 5.10 (4.80–6.30)	(10) 5.30 (4.70–6.00)
Non-HDL cholesterol ^a^ (N_missing_) Median (IQR)	(2) 3.60 (2.99–4.26)	(0) 3.71 (3.04–4.29)	(0) 3.72 (3.11–4.46)	(1) 3.97 (3.40–5.03)	(10) 3.82 (3.17–4.56)
HDL cholesterol ^a^ (N_missing_) Median (IQR)	(2) 1.39 (1.17–1.64)	(0) 1.45 (1.16–1.76)	(0) 1.51 (1.30–1.75)	(1) 1.29 (1.09–1.47)	(10) 1.43 (1.18–1.74)
Triglycerides ^a^ (N_missing_) Median (IQR)	(3) 0.95 (0.68–1.51)	(0) 1.11 (0.80–1.48)	(0) 0.99 (0.73–1.20)	(1) 1.32 (1.06–1.92)	(10) 0.99 (0.74–1.41)
Glucose and insulin	CF N = 240 ^f^	CWP N = 170 ^f^	IB N = 97 ^f^	MCS N = 26 ^f^	No FSS N = 4017 ^f^
Glucose ^a^ (N_missing_) Median (IQR)	(4) 5.30 (4.90–5.60)	(1) 5.50 (5.10–6.00)	(0) 5.20 (4.90–5.70)	(2) 5.45 (5.10–6.35)	(23) 5.40 (5.10–5.80)
HbA1c ^b^ (N_missing_) Median (IQR)	(1) 5.40 (5.10–5.60)	(0) 5.50 (5.20–5.70)	(0) 5.30 (5.20–5.60)	(1) 5.50 (5.30–5.80)	(20) 5.40 (5.20–5.60)
Insulin ^c^ (N_missing_) Median (IQR)	(14) 51.2 (31.0–72.3)	(5) 52.7 (35.6–78.3)	(6) 45.8 (30.8–70.1)	(2) 57.7 (40.8–112)	(183) 46.3 (31.8–67.9)
HOMA-IR (N_missing_) Median (IQR)	(15) 1.70 (1.00–2.55)	(6) 1.85 (1.21–2.94)	(6) 1.53 (0.98–2.60)	(2) 2.10 (1.36–4.75)	(191) 1.61 (1.07–2.47)

HDL: high-density lipoprotein. HbA1c: % glycated hemoglobin; HOMA-IR: homeostatic model assessment for insulin resistance ((plasma insulin × plasma glucose/22.5) × 0.144). ^a^: mmol/L; ^b^: % glycated hemoglobulin; ^c^: pmol/L; ^d^: number of persons with no FSS at follow-up and where information on all FSSs is present; ^e^: only participants with information on lipid-lowering medications; ^f^: only participants with information on glucose-lowering medications.

**Table 3:** Total cholesterol, non-HDL cholesterol, HDL cholesterol, triglycerides, glucose, HbA1c, insulin, and HOMA-IR at baseline among fasting participants as possible risk factors for the development of bodily distress syndrome (BDS) (total, single-organ, and multi-organ) in a five-year period. The DanFunD study.

Lipids	Total BDS		Single-Organ BDS		Multi-Organ BDS
	Model 1 OR (95% CI)	Model 3 OR (95% CI)	Model 1 OR (95% CI)	Model 3 OR (95% CI)	Model 1 ^a^ OR (95% CI)
Total cholesterol	***0.989 (0.979–0.999)***	***0.982 (0.971–0.993)***	***0.988 (0.978–0.998)***	***0.981 (0.970–0.992)***	1.000 (0.961–1.041)
Non-HDL cholesterol	0.998 (0.988–1.008)	***0.986 (0.976–0.998)***	0.997 (0.987–1.007)	***0.985 (0.974–0.997)***	1.013 (0.975–1.053)
HDL cholesterol	***0.946 (0.923–0.970)***	***0.968 (0.941–0.997)***	***0.948 (0.924–0.973)***	***0.970 (0.942–0.999)***	0.919 (0.827–1.022)
Triglycerides	**1.012 (1.000–1.024)**	0.990 (0.975–1.006)	1.012(1.000–1.024)	0.990 (0.975–1.006)	1.014 (0.970–1.059)
Glucose and insulin					
Glucose	1.016 (0.896–1.152)	0.900 (0.768–1.054)	1.012 (0.889–1.152)	0.888 (0.754–1.045)	1.081 (0.708–1.650)
HbA1c	1.139 (0.906–1.433)	0.990 (0.761–1.289)	1.131 (0.893–1.431)	0.985 (0.753–1.289)	1.266 (0.576–2.784)
Insulin	***1.002 (1.001–1.004)***	1.001 (1.000–1.002)	***1.002 (1.001–1.004)***	1.001 (1.000–1.002)	1.000 (0.992–1.009)
HOMA-IR	**1.004 (1.001–1.007)**	1.001 (0.999–1.004)	**1.004 (1.001–1.007)**	1.002 (0.999–1.004)	1.000 (0.981–1.019)

Model 1: Adjusted for age, sex, intake of cholesterol-lowering medications (only in analyses of lipids) and intake of glucose-lowering medications (only in analyses of glucose and insulin). Model 3: Further adjusted for BMI, subjective social status, lifestyle factors (smoking, alcohol intake, diet, and physical activity), and sleep quality; ^a^: too few cases to adjust for BMI, social factors, lifestyle, and sleep patterns. HDL: high-density lipoprotein; HbA1c: % glycated hemoglobin; HOMA-IR: homeostatic model assessment for insulin resistance; OR: odds ratio; CI: confidence interval. Lipids and HOMA-IR were analyzed per 0.1 unit, and glucose, HbA1c and insulin per 1 unit. Numbers in italics and bold indicate significance.

**Table 4:** Total cholesterol, non-HDL cholesterol, HDL cholesterol, triglycerides, glucose, HbA1c, insulin, and HOMA at baseline among fasting participants as possible risk factors for the development of chronic fatigue (CF) in a five-year period. The DanFunD study.

Lipids	CF	
	Model 1 OR (95% CI)	Model 3 OR (95% CI)
Total cholesterol	0.986 (0.971–1.001)	***0.980 (0.964–0.996)***
Non-HDL cholesterol	0.994 (0.980–1.009)	0.985 (0.970–1.002)
HDL cholesterol		
Women	0.979 (0.939–1.022)	0.991 (0.946–1.038)
Men	***0.874 (0.808–0.945)***	***0.893 (0.821–0.971)***
Triglycerides		
<1.0 mmol/L	0.950 (0.876–1.029)	0.916 (0.837–1.003)
≥1.0 mmol/L	***1.031 (1.014–1.049)***	***1.022 (1.003–1.041)***
Glucose and insulin		
Glucose		
<5.4	***0.621 (0.411–0.939)***	***0.599 (0.382–0.938)***
≥5.4	1.058 (0.854–1.310)	0.993 (0.761–1.295)
HbA1c	1.169 (0.833–1.640)	1.109 (0.758–1.621)
Insulin		
<23	***0.933 (0.883–0.985)***	***0.925 (0.871–0.981)***
23–104	***1.007 (1.002–1.013)***	1.002 (0.995–1.009)
≥105	***1.002 (1.000–1.004)***	***1.002 (1.000–1.004)***
HOMA-IR		
<0.8	***0.842 (0.734–0.966)***	***0.829 (0.713–0.964)***
0.8–3.9	***1.017 (1.003–1.032)***	1.003 (0.985–1.021)
≥4.0	1.003 (1.000–1.006)	1.003 (1.000–1.006)

Model 1: Adjusted for age, sex, intake of cholesterol-lowering medications (only in analyses of lipids) and intake of glucose-lowering medications (only in analyses of glucose and insulin). Model 3: Further adjusted for BMI, subjective social status, lifestyle factors (smoking, alcohol intake, diet, and physical activity), and sleep quality. HDL: high-density lipoprotein; HbA1c: % glycated hemoglobin; HOMA-IR: homeostatic model assessment for insulin resistance; OR: odds ratio; CI: confidence interval. Lipids and HOMA-IR were analyzed per 0.1 unit, and glucose, HbA1c and insulin per 1 unit. Numbers in italics and bold indicate significance.

**Table 5:** Total cholesterol, non-HDL cholesterol, HDL cholesterol, triglycerides, glucose, HbA1c, insulin, and HOMA-IR at baseline among fasting participants as possible risk factors for the development of chronic widespread pain (CWP) in a five-year period. The DanFunD study.

Lipids	CWP	
	Model 1 OR (95% CI)	Model 3 OR (95% CI)
Total cholesterol	***0.982 (0.966–0.999)***	0.982 (0.964–1.001)
Non-HDL cholesterol	0.990 (0.974–1.007)	0.985 (0.967–1.003)
HDL cholesterol	0.960 (0.922–1.000)	0.986 (0.942–1.032)
Triglycerides	1.014 (0.996–1.033)	1.002 (0.980–1.024)
Glucose and insulin		
Glucose	1.079 (0.920–1.265)	1.042 (0.869–1.251)
HbA1c	1.108 (0.791–1.552)	0.940 (0.633–1.394)
Insulin	1.000 (0.999–1.002)	0.999 (0.995–1.002)
HOMA-IR	1.001 (0.998–1.003)	0.999 (0.995–1.003)

Model 1: Adjusted for age, sex, intake of cholesterol-lowering medications (only in analyses of lipids) and intake of glucose-lowering medications (only in analyses of glucose and insulin). Model 3: Further adjusted for lifestyle factors (smoking, alcohol intake, diet, and physical activity), and sleep quality. HDL: high-density lipoprotein; HbA1c: % glycated hemoglobin; HOMA-IR: homeostatic model assessment for insulin resistance; OR: odds ratio; CI: confidence interval. Lipids and HOMA-IR were analyzed per 0.1 unit, and glucose, HbA1c and insulin per 1 unit. Numbers in italics and bold indicate significance.

**Table 6:** Total cholesterol, non-HDL cholesterol, HDL cholesterol, triglycerides, glucose, HbA1c, insulin, and HOMA-IR at baseline among fasting participants as possible risk factors for the development of irritable bowel (IB) in a five-year period. The DanFunD study.

Lipids	IB	
	Model 1 OR (95% CI)	Model 2 ^a^ OR (95% CI)
Total cholesterol	1.017 (0.996–1.040)	1.022 (1.000–1.045)
Non-HDL cholesterol	1.017 (0.995–1.038)	***1.024 (1.002–1.047)***
HDL cholesterol	1.001 (0.950–1.055)	0.990 (0.936–1.048)
Triglycerides	1.002 (0.969–1.036)	1.006 (0.973–1.040)
Glucose and insulin		
Glucose	0.902 (0.635–1.282)	0.949 (0.668–1.347)
HbA1c	1.157 (0.672–1.993)	1.167 (0.658–2.072)
Insulin	1.001 (0.998–1.004)	1.001 (0.998–1.003)
HOMA-IR	1.001 (0.996–1.006)	1.001 (0.996–1.006)

Model 1: Adjusted for age, sex, intake of cholesterol-lowering medications (only in analyses of lipids) and intake of glucose-lowering medications (only in analyses of glucose and insulin). Model 2: Further adjusted for BMI and subjective social status. ^a^: Model 2 plus sleep quality. HDL: high-density lipoprotein; HbA1c: % glycated hemoglobin; HOMA-IR: homeostatic model assessment for insulin resistance; OR: odds ratio; CI: confidence interval. Lipids and HOMA-IR were analyzed per 0.1 unit, and glucose, HbA1c and insulin per 1 unit. Numbers in italics and bold indicate significance.

**Table 7:** Total cholesterol, non-HDL cholesterol, HDL cholesterol, triglycerides, glucose, HbA1c, insulin, and HOMA at baseline among fasting participants as possible risk factors for the development of multiple chemical sensitivity (MCS) in a five-year period. The DanFunD study.

Lipids	MCS
	Model 1 ^a^ OR (95% CI)
Total cholesterol	1.005 (0.963–1.048)
Non-HDL cholesterol	1.027 (0.988–1.066)
HDL cholesterol	***0.849 (0.754–0.957)***
Triglycerides	
<1.0 mmol/L	***1.701 (1.078–2.683)***
≥1.0 mmol/L	1.021 (0.982–1.061)
Glucose and insulin	
Glucose	***1.352 (1.096–1.669)***
HbA1c	1.458 (0.787–2.702)
Insulin	1.001 (0.999–1.003)
HOMA-IR	1.002 (0.999–1.005)

Model 1: Adjusted for age, sex, intake of cholesterol-lowering medications (only in analyses of lipids) and intake of glucose-lowering medications (only in analyses of glucose and insulin). ^a^: too few cases to adjust for BMI, social position, lifestyle, and sleep pattern. HDL: high-density lipoprotein; HbA1c: % glycated hemoglobin; HOMA-IR: homeostatic model assessment for insulin resistance; OR: odds ratio; CI: confidence interval. Lipids and HOMA-IR were analyzed per 0.1 unit, and glucose, HbA1c and insulin per 1 unit. Numbers in italics and bold indicate significance.

## Data Availability

Restrictions apply to the availability of these data according to Danish law. Requests for access to the data need approval from appropriate Danish authorities and are subject to Danish regulations on personal data protection. A request for the arrangement of data transfer agreements can be sent to the corresponding author or ckff@regionh.dk.

## Supplementary Materials

The following supporting information can be downloaded at: https://www.mdpi.com/article/10.3390/jcm15155793/s1, Table S1. Distribution of total cholesterol, non-HDL cholesterol, HDL-cholesterol, triglycerides, glucose, HbA1c, insulin, and HOMA-IR at baseline in persons who adhered to fasting and who developed functional somatic syndromes (FSS) without co-morbid FSS (chronic fatigue (CF), chronic widespread pain (CWP), irritable bowel (IB), and multiple chemical sensitivity (MCS)) and not (no FSS) at follow-up. Only persons without FSS at baseline were included (N = 4557). Thus, the numbers represent the cumulative incidence of various FSS. The DanFunD study. Table S2. Total cholesterol, non-HDL cholesterol, HDL-cholesterol, triglycerides, glucose, HbA1c, insulin, and HOMA at baseline among fasting participants as possible risk factors for the development of chronic fatigue (CF) without comorbid functional somatic syndromes (FSS) in a five-year period. The DanFunD study. Table S3. Total cholesterol, non-HDL cholesterol, HDL-cholesterol, triglycerides, glucose, HbA1c, insulin, and HOMA-IR at baseline among fasting participants as possible risk factors for the development of chronic widespread pain (CWP) without comorbid functional somatic syndrome (FSS) in a five-year period. The DanFunD study. Table S4. Total cholesterol, non-HDL cholesterol, HDL-cholesterol, triglycerides, glucose, HbA1c, insulin, and HOMA-IR at baseline among fasting participants as possible risk factors for the development of irritable bowel (IB) without comorbid functional somatic syndrome (FSS) in a five-year period. The DanFunD study. Table S5. Total cholesterol, non-HDL cholesterol, HDL-cholesterol, triglycerides, glucose, HbA1c, insulin, and HOMA at baseline among fasting participants as possible risk factors for the development of multiple chemical sensitivity (MCS) without comorbid functional somatic syndromes (FSS) in a five-year period. The DanFunD study.
