# Supplementary material for: Insulin, Glucose and Lipid Biomarkers as Possible Risk Factors for Functional Somatic Disorder: A Five-Year Follow-Up of the DanFunD Cohort
Source: J Clin Med. 2026 Jul 24;15(15):5793. doi: 10.3390/jcm15155793 (PMC13466561; doi:10.3390/jcm15155793)
Supplement: Supplementary file 1 [file jcm-15-05793-s001.zip › jcm-4386416-supplementary.pdf]

Supplementary Materials

Jørgensen T, Jacobsen RK, Jørgensen SW, Petersen MW, Bjerregaard AA, Gormsen LK, Carstensen TBW, Schovsbo SU, Kårhus LL, Jørgensen NR, Linneberg A, Dantoft TM. Insulin, glucose and lipid biomarkers as possible Risk Factors for Functional Somatic Disorder. Five-Year Follow-up of the DanFunD Cohort.

Table S1. Distribution of total cholesterol, non-HDL cholesterol, HDL-cholesterol, triglycerides, glucose, HbA1c, insulin, and HOMA-IR at baseline in persons who adhered to fasting and who developed functional somatic syndromes (FSS) without co-morbid FSS (chronic fatigue (CF), chronic widespread pain (CWP), irritable bowel (IB), and multiple chemical sensitivity (MCS)) and not (no FSS) at follow-up. Only persons without FSS at baseline were included (N=4,557). Thus, the numbers represent the cumulative incidence of various FSS. The DanFunD study.

**Lipid metabolism**	CF pure[d]<br>N=191	CWP pure[d]<br>N=131	IB pure[d]<br>N=73	MCS pure[d]<br>N=18	No FSS [c,d]<br>N = 4.018
**Cholesterol[a] ($N_{missing}$)**<br>***Median (IQR)***	(2)<br>5.00 (4.40-5.60)	(0)<br>5.20 (4.50-6.00)	(0)<br>5.00 (4.60-6.00)	(1)<br>5.30 (4.90-6.30)	(10)<br>5.30 (4.70-6.00)
**Non-HDL cholesterol[a] ($N_{missing}$)**<br>***Median (IQR)***	(2)<br>3.58 (2.81-4.23)	(0)<br>3.78 (3.03-4.44)	(0)<br>3.63 (3.07-4.41)	(1)<br>3.97 (3.40-5.11)	(10)<br>3.82 (3.17-4.56)
**HDL cholesterol[a] ($N_{missing}$)**<br>***Median (IQR)***	(2)<br>1.37 (1.15-1.61)	(0)<br>1.44 (1.10-1.73)	(0)<br>1.54 (1.30-1.75)	(1)<br>1.28 (1.09-1.38)	(10)<br>1.43 (1.18-1.74)
**Triglycerides[a] ($N_{missing}$)**<br>***Median (IQR)***	(3)<br>0.93 (0.66-1.53)	(0)<br>1.16 (0.85-1.52)	(0)<br>0.95 (0.73-1.19)	(1)<br>1.32 (1.06-1.85)	(10)<br>0.99 (0.74-1.41)
**Glucose metabolism**	CF pure[e]<br>N=192	CWP pure[e]<br>N=130	IB pure[e]<br>N=73	MCS pure[e]<br>N=18	No FSS[c,e]<br>N=4,017
**Glucose[a] ($N_{missing}$)**<br>***Median (IQR)***	(3)<br>5.30 (4.90-5.60)	(1)<br>5.50 (5.20-6.10)	(0)<br>5.30 (5.00-5.70)	(1)<br>5.70 (5.10-6.30)	(23)<br>5.40 (5.10-5.80)
**HbA1c ($N_{missing}$)**<br>***Median (IQR)***	(1)<br>5.40 (5.10-5.60)	(0)<br>5.50 (5.20-5.70)	(0)<br>5.30 (5.20-5.60)	(1)<br>5.50 (5.30-5.80)	(19)<br>5.40 (5.20-5.60)
**Insulin[b] ($N_{missing}$)**<br>***Median (IQR)***	(12)<br>50.8 (30.9-74.7)	(4)<br>53.4 (38.7-83.0)	(6)<br>45.7 (30.8-70.5)	(1)<br>60.0 (40.6-119)	(181)<br>46.3 (31.8-67.9)
**HOMA-IR ($N_{missing}$)**<br>***Median (IQR)***	(13)<br>1.64 (0.95-2.72)	(5)<br>1.95 (1.25-3.13)	(6)<br>1.50 (0.98-2.71)	(1)<br>2.04 (1.32-4.81)	(189)<br>1.61 (1.07-2.47)

Pure: Without co-morbid FSS.

HDL: High Density Lipoprotein. HbA1c: % glycated hemoglobin; HOMA-IR: Homeostatic model assessment for insulin resistance ((plasma insulin x plasma glucose/22.5) X 0.144)

a: mmol/l; b: pmol/l; c: number of persons with no FSS at follow-up and where information on all FSS is present; d: only participants with information on lipid lowering medications; e: only participants with information on glucose lowering medications.

Table S2. Total cholesterol, non-HDL cholesterol, HDL-cholesterol, triglycerides, glucose, HbA1c, insulin, and HOMA at baseline among fasting participants as possible risk factors for the development of chronic fatigue (CF) without comorbid functional somatic syndromes (FSS) in a five-year period. The DanFunD study.

Lipids	CF without comorbid FSS	
	Model 1<br>OR (95 % CI)	Model 3<br>OR (95 % CI)
**Total cholesterol**	***0.980 (0.964-0.997)***	***0.973 (0.965-0.992)***
**Non-HDL cholesterol**	0.992 (0.975-1.008)	***0.981 (0.962-0.999)***
**HDL cholesterol**		
**Women**	0.968 (0.922-1.016)	0.981 (0.930-1.036)
**Men**	***0.859 (0.789-0.935)***	***0.878 (0.802-0.963)***
**Triglycerides**		
**< 1.0 mmol/l**	0.919 (0.841-1.004)	***0.872 (0.790-0.963)***
**≥1.0 mmol/l**	***1.034 (1.016-1.053)***	***1.026 (1.006-1.045)***
Glucose and insulin		
**Glucose**		
<5.4	***0.517 (0.332-0.805)***	***0.494 (0.302-0.807)***
≥5.4	1.072(0.848-1.356)	1.026 (0.775-1.359)
**HbA1c**	1.226 (0.856-1.758)	1.160 (0.771-1.748)
**Insulin**		
<23	***0.935 (0.878-0.995)***	***0.922 (0.864-0.985)***
23-104	***1.007 (1.001-1.013)***	1.002 (0.994-1.010)
**≥105**	***1.002 (1.000 1.004)***	***1.002 (1.000-1.004)***
**HOMA-IR**		
**<0.8**	***0.848 (0.725 - 0.991)***	***0.825 (0.699 - 0.973)***
**0.8-3.9**	***1.016 (1.000 - 1.033)***	1.002 (0.982 - 1.023)

**≥4.0**	1.004 (1.000 - 1.008)	1.004 (1.000 - 1.007)

Model 1: Adjusted for age, sex, and intake of cholesterol lowering medications (in analyses of lipid parameters) and intake of glucose lowering medications (in analyses of glucose and insulin parameters).

Model 3: Further adjusted for BMI, subjective social status, lifestyle factors (smoking, alcohol intake, diet, and physical activity), and sleep quality

HDL: High Density Lipoprotein; HbA1c: % glycated hemoglobin; HOMA-IR: Homeostatic model assessment for insulin resistance ((plasma insulin x plasma glucose/22.5) X 0.144); OR: Odds ratio; CI: Confidence Interval

Lipids and HOMA-IR were analyzed per 0.1 unit, and glucose, HbA1c and insulin per 1 unit. Numbers in italic and bold indicate significance.

Table S3. Total cholesterol, non-HDL cholesterol, HDL-cholesterol, triglycerides, glucose, HbA1c, insulin, and HOMA-IR at baseline among fasting participants as possible risk factors for the development of chronic widespread pain (CWP) without comorbid functional somatic syndrome (FSS) in a five-year period. The DanFunD study.

Lipids	CWP without comorbid FSS	
	Model 1<br>OR (95 % CI)	Model 2[a]<br>OR (95 % CI)
**Total cholesterol**		
**Women**	1.001 (0.977-1.026)	0.993 (0.968-1.018)
**Men**	***0.963 (0.936-0.990)***	***0.963 (0.937-0.990)***
**Non-HDL cholesterol**	0.993 (0.974-1.012)	0.981 (0.962-1.001)
**HDL-cholesterol**		
**Women**	0.984 (0.931-1.041)	1.020 (0.963-1.081)
**Men**	***0.890 (0.818-0.968)***	0.919 (0.843-1.001)
**Triglycerides**	1.016 (0.997-1.036)	1.000 (0.976-1.025)
Glucose and insulin		
**Glucose**	0.997 (0.811-1.227)	0.899 (0.706-1.146)
**HbA1c**	1.047 (0.716-1.530)	0.901 (0.591-1.372)
**Insulin**		
**<23**	1.053 (0.917-1.209)	1.038 (0.905-1.191)
**23-103**	***1.011 (1.003-1.019)***	1.003 (0.994-1.012)
**≥104**	0.992 (0.980-1.003)	0.990 (0.979-1.002)
**HOMA-IR**		
**<0.8**	1.187 (0.838-1.681)	1.131 (0.799-1.599)
**0.8-3.9**	***1.020 (1.001-1.040)***	1.000 (0.979-1.022)
**≥4.0**	0.992 (0.975-1.008)	0.991 (0.975-1.007)

Model 1: Adjusted for age, sex, and intake of cholesterol lowering medications (in analyses of lipid parameters) and intake of glucose lowering medications (in analyses of glucose and insulin parameters).

Model 2: Further adjusted for BMI and subjective social status. a: Model 2 was further adjusted for sleep quality

HDL: High Density Lipoprotein; HbA1c: % glycated hemoglobin; HOMA-IR: Homeostatic model assessment for insulin resistance ((plasma insulin x plasma glucose/22.5) X 0.144); OR: Odds ratio; CI: Confidence Interval

Lipids and HOMA-IR were analyzed per 0.1 unit, and glucose, HbA1c and insulin per 1 unit. Numbers in italic and bold indicate significance.

Table S4. Total cholesterol, non-HDL cholesterol, HDL-cholesterol, triglycerides, glucose, HbA1c, insulin, and HOMA-IR at baseline among fasting participants as possible risk factors for the development of irritable bowel (IB) without comorbid functional somatic syndrome (FSS) in a five-year period. The DanFunD study.

Lipids	IB without comorbid FSS	
	Model 1<br>OR (95 % CI)	Model 2[a]<br>OR (95 % CI)
**Total cholesterol**	1.015 (0.990-1.041)	1.017 (0.992-1.044)
**Non-HDL cholesterol**	1.015 (0.990-1.040)	1.022 (0.996-1.048)
**HDL cholesterol**	0.999 (0.941-1.061)	0.976 (0.915-1.042)
**Triglycerides**	0.996 (0.956-1.038)	1.002 (0.961-1.045)
Glucose and insulin		
**Glucose**	0.899 (0.588-1.373)	0.907 (0.584-1.408)
**HbA1c**	1.334 (0.771-2.309)	1.404 (0.792-2.489)
**Insulin**	1.003 (0.998-1.007)	1.004 (0.999-1.008)
**HOMA-IR**	1.005 (0.995-1.014)	1.006 (0.996-1.015)

Model 1: Adjusted for age, sex, and intake of cholesterol lowering medications (in analyses of lipid parameters) and intake of glucose lowering medications (in analyses of glucose and insulin parameters).

Model 2: Further adjusted for BMI and subjective social status. a: Model 2 was further adjusted for sleep quality.

HDL: High Density Lipoprotein; HbA1c: % glycated hemoglobin; HOMA-IR: Homeostatic model assessment for insulin resistance ((plasma insulin x plasma glucose/22.5) X 0.144); OR: Odds ratio; CI: Confidence Interval

Lipids and HOMA-IR were analyzed per 0.1 unit, and glucose, HbA1c and insulin per 1 unit. Numbers in italic and bold indicate significance.

Table S5. Total cholesterol, non-HDL cholesterol, HDL-cholesterol, triglycerides, glucose, HbA1c, insulin, and HOMA at baseline among fasting participants as possible risk factors for the development of multiple chemical sensitivity (MCS) without comorbid functional somatic syndromes (FSS) in a five-year period. The DanFunD study.

Lipids	MCS without co-morbid FSS
	Model 1[a]<br>OR (95 % CI)
**Total cholesterol**	1.008 (0.963-1.054)
**Non-HDL cholesterol**	1.026 (0.982-1.072)
**HDL cholesterol**	***0.865 (0.752-0.995)***
**Triglycerids**	1.029 (0.996-1.063)
Glucose and insulin	
**Glucose**	1.157 (0.762-1.756)
**HbA1c**	1.352 (0.587-3.115)
**Insulin**	1.004 (0.999-1.009)
**HOMA-IR**	1.006 (0.997-1.015)

Model 1: Adjusted for age, sex, and intake of cholesterol lowering medications (in analyses of lipid parameters) and intake of glucose lowering medications (in analyses of glucose and insulin parameters).

a: Too few cases to adjust for BMI, social position, lifestyle, and sleep pattern.

HDL: High Density Lipoprotein; HbA1c: % glycated hemoglobin; HOMA-IR: Homeostatic model assessment for insulin resistance ((plasma insulin x plasma glucose/22.5) X 0.144); OR: Odds ratio; CI: Confidence Interval

Lipids and HOMA-IR were analyzed per 0.1 unit, and glucose, HbA1c and insulin per 1 unit. Numbers in italic and bold indicate significance.
